# Quantitative Biomarkers Derived from a Novel, Contrast-Free Ultrasound, High-Definition Microvessel Imaging for Differentiating Choroidal Tumors

**DOI:** 10.3390/cancers16020395

**Published:** 2024-01-17

**Authors:** Shaheeda A. Adusei, Soroosh Sabeti, Nicholas B. Larson, Lauren A. Dalvin, Mostafa Fatemi, Azra Alizad

**Affiliations:** 1 Department of Physiology and Biomedical Engineering, Mayo Clinic College of Medicine and Science, 200 1st St. SW, Rochester, MN 55905, USAfatemi.mostafa@mayo.edu (M.F.); 2 Department of Quantitative Health Sciences, Mayo Clinic College of Medicine and Science, 200 1st St. SW, Rochester, MN 55905, USA; 3 Department of Ophthalmology, Mayo Clinic College of Medicine and Science, 200 1st St. SW, Rochester, MN 55905, USA; 4 Department of Radiology, Mayo Clinic College of Medicine and Science, 200 1st St. SW, Rochester, MN 55905, USA

**Keywords:** choroidal melanoma, ocular tumors, ultrasound microvessel imaging, flow imaging, vessel morphological biomarkers

## Abstract

**Simple Summary:**

Accurate diagnosis of early-stage choroidal melanoma, the most commonly occurring malignancy of the eye, is extremely important yet highly challenging. Unfortunately, due to challenges in obtaining adequate sampling and the risk of vision loss, intraocular melanoma remains largely a clinical diagnosis, which can be subjective. Most of the existing tools and diagnostic methods lack definitive discriminative features, leading to uncertainties in clinical diagnoses. In this work, we present and evaluate a novel, contrast-free ultrasound-based method for quantitative assessment of microvascular characteristics of choroidal tumors, aiming at distinguishing malignant lesions based on the complex and irregular microvessel formations within them. Using this method, we visualize intratumoral microvascular networks, estimate their morphological features using objective quantitative metrics, and perform statistical analyses demonstrating differences between lesions based on their malignancy status. The results of this preliminary study show promise for further assessment of the method as a complementary diagnostic tool for ocular tumors.

**Abstract:**

Angiogenesis has an essential role in the de novo evolution of choroidal melanoma as well as choroidal nevus transformation into melanoma. Differentiating early-stage melanoma from nevus is of high clinical importance; thus, imaging techniques that provide objective information regarding tumor microvasculature structures could aid accurate early detection. Herein, we investigated the feasibility of quantitative high-definition microvessel imaging (qHDMI) for differentiation of choroidal tumors in humans. This new ultrasound-based technique encompasses a series of morphological filtering and vessel enhancement techniques, enabling the visualization of tumor microvessels as small as 150 microns and extracting vessel morphological features as new tumor biomarkers. Distributional differences between the malignant melanomas and benign nevi were tested on 37 patients with choroidal tumors using a non-parametric Wilcoxon rank-sum test, and statistical significance was declared for biomarkers with *p*-values < 0.05. The ocular oncology diagnosis was choroidal melanoma (malignant) in 21 and choroidal nevus (benign) in 15 patients. The mean thickness of benign and malignant masses was 1.70 ± 0.40 mm and 3.81 ± 2.63 mm, respectively. Six HDMI biomarkers, including number of vessel segments (*p* = 0.003), number of branch points (*p* = 0.003), vessel density (*p* = 0.03), maximum tortuosity (*p* = 0.001), microvessel fractal dimension (*p* = 0.002), and maximum diameter (*p* = 0.003) exhibited significant distributional differences between the two groups. Contrast-free HDMI provided noninvasive imaging and quantification of microvessels of choroidal tumors. The results of this pilot study indicate the potential use of qHDMI as a complementary tool for characterization of small ocular tumors and early detection of choroidal melanoma.

## 1. Introduction

Uveal melanoma is the most common primary intraocular malignancy in adults, with 90% occurrence in choroid. These malignancies can arise from preexisting choroidal nevus or de novo [1,2]. Distinguishing early-stage choroidal melanoma from nevus can be challenging as they share similar ophthalmoscopic appearances when small [3]. Heretofore, no single modality can successfully discriminate between these two entities. Biopsy for histopathologic diagnosis is often unhelpful due to challenges in obtaining adequate sampling and the risk of vision loss [4,5]. Consequently, multimodal imaging—including ultrasound (US), optical coherence tomography (OCT), OCT-angiography (OCTA), and fundus autofluorescence (FA)—is the most frequently employed diagnostic approach, but its interpretation still requires the clinical judgement of an expert ocular oncologist [6]. Furthermore, diagnosis remains somewhat subjective.

Angiogenesis is essential to the development and progression of uveal melanoma [7]. Malignant transformation of an existing nevus is also accompanied by the formation of new, aberrant, and structurally abnormal vessels [8,9]. Currently, no imaging modality can directly assess tumor angiogenesis-related morphological changes as biomarkers for cancer detection, as the available imaging modalities do not have the sensitivity or resolution to characterize tumor microvessel structural abnormalities.

The report of the first experience of using echo color Doppler with an echographic contrast agent demonstrated the enhancement of Doppler signals in ocular tumors in different degrees [10]. Color Doppler ultrasound is helpful for differentiating a solid tumor from retinal detachment; however, it is not sensitive to slow flows and does not reveal tumor microvessels [11]. The limitations of OCTA include motion artifacts and the need for extensive image processing. Clinical studies using contrast-enhanced ultrasound [12] and preclinical studies of super-resolution ultrasound and ultrasound localization microscopy (ULM) [13] are ongoing research areas for the detection of choroidal melanoma. However, none of these methods offer quantification of tumor microvessel morphology. Furthermore, careful use of ultrasound contrast agents is warranted to avoid damage to the ocular vasculature [14].

To address these research gaps, a contrast-free ultrasound-based technology named quantitative high-definition microvasculature imaging (qHDMI) was developed to visualize microvessels as small as 150 µm [15]. This technique is equipped with a series of image processing operations, morphological filtering, and vessel enhancement to extract and quantify vessel morphological parameters as quantitative vessel biomarkers [15,16,17] and has been tested for diagnosis of various cancers [18,19,20,21,22,23].

The present work comprises an assessment framework to differentiate choroidal melanoma from a choroidal nevus. We hypothesize that the proposed contrast-free qHDMI provides quantitative biomarker information regarding morphological features of tumor microvessels and has the potential to objectively classify early-stage small choroidal melanomas from benign nevi, thus rendering this method operator-independent and eliminating the observer/reader variability for reliable clinical use.

## 2. Materials and Methods

### 2.1. Ethical Statement

This prospective study was Health Insurance Portability and Accountability Act–compliant and approved by the institutional review board (IRB#22-001824). Each participant signed an IRB-approved written informed consent with permission for publication.

### 2.2. Participants

**Figure 1:**
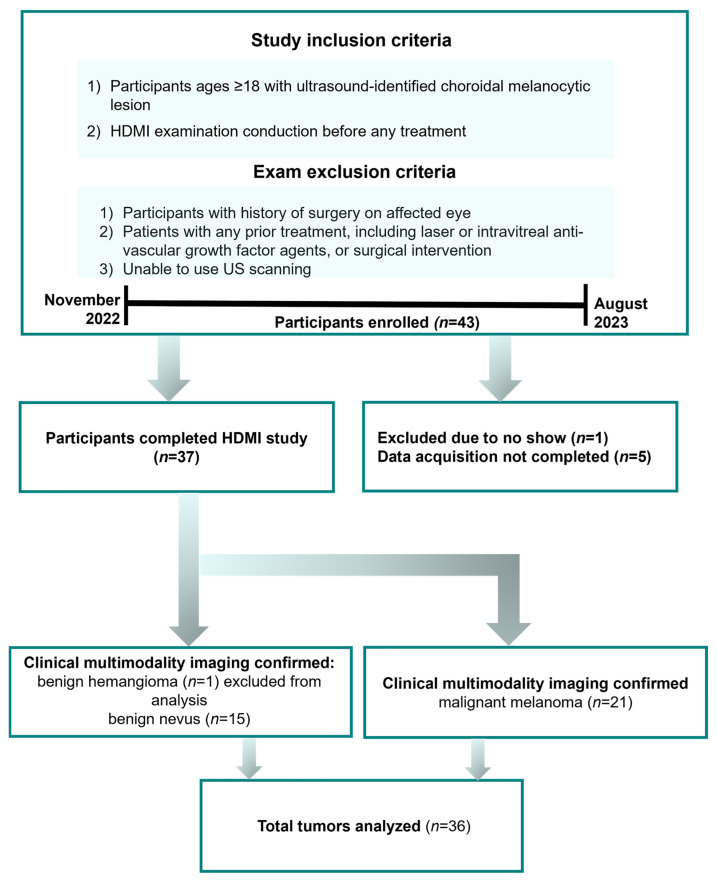
Flowchart for the participants enrolled in this study.

This pilot study consisted of 36 participants with choroidal melanocytic lesions recruited consecutively from November 2022 through August 2023. The inclusion and exclusion criteria are detailed in the study flow chart shown in Figure 1. The investigative team was blinded to the clinical assessments during the analysis, and the diagnosing ocular oncologist was blinded to the imaging results until after the diagnosis was rendered. Clinical assessment by a fellowship-trained ocular oncologist with multimodal ophthalmic imaging served as the diagnostic reference standard. All participants completed our research imaging examination before any treatment. Figure 1 indicates the flow diagram of the participants enrolled in this study.

### 2.3. Clinical Multimodal Imaging

All participants enrolled in this study had clinical multimodal imaging examinations, including conventional ultrasonography, fundus autofluorescence (FA), OCT, and OCTA, as part of their clinical care. Risk factors predictive of the transformation of a choroidal nevus into melanoma were clinically considered [6]. These risk factors include tumor thickness > 2 mm, basal diameter > 5 mm, subretinal fluid, symptoms of visual acuity loss to 20/50 or worse, orange pigment, ultrasonographic features such as hollowness or low internal reflectivity, and largest basal diameter greater than 5 mm. Choroidal melanoma was diagnosed clinically at initial presentation if large with features of acoustic hollowness and subretinal fluid, or, for small lesions, was diagnosed after documented growth of 0.1 mm or greater within a period of 6 months or less. Patients with a choroidal nevus had documented stability of at least 1 year prior to study inclusion.

### 2.4. High-Definition Microvasculature Imaging and Vessel Extraction

All imaging studies were performed using a research ultrasound platform, Verasonics Vantage 128 scanner equipped with a linear array transducer, L22vXLF with a center frequency of 16.5MHz (Verasonics, Inc., Kirkland, WA, USA). Participants were scanned while seated in a reclining examination chair. Two sonographers with more than 10 years of experience conducted the research US study. Using Genteal gel over the eyelid, the ultrasound transducer was placed over the eyelid and participants were asked to look in a direction that allowed for the best visualization of the tumor on the B-scan. Ocular lesions were first identified using plane-wave B-mode ultrasound. Ultrafast ultrasound imaging via 3-angle coherent plane-wave compounding was performed at an effective frame rate of 1000 Hz over a one-second time span [24]. No contrast-enhancing agent was used. Data were downloaded from the machine and all processing was performed offline using MATLAB, RRID:SCR_001622 (Mathworks, Natick, MA). Microvasculature images were generated through post-processing of the data using a series of clutter filtering, denoising, and vessel enhancement steps [15,25].

### 2.5. HDMI Quantification and Quantitative Biomarkers

**Figure 2:**
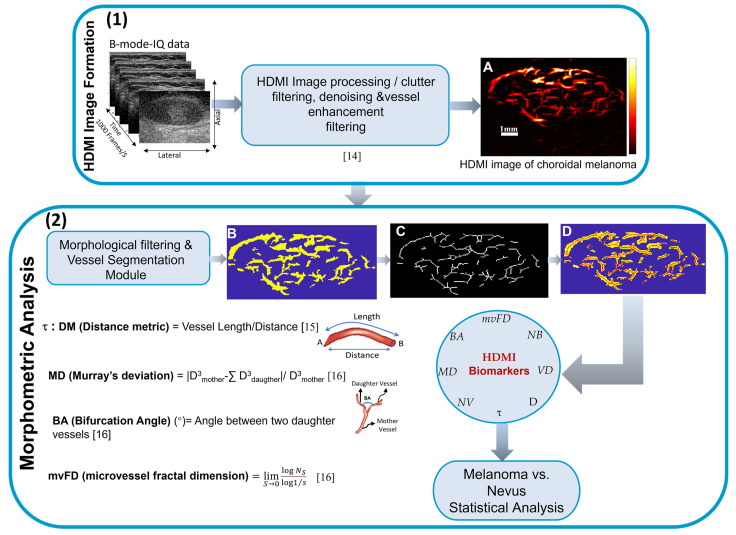
HDMI image formation and processing steps to prepare HDMI image for quantification: (1) Microvasculature image formation of a malignant choroidal melanoma processing steps; (**A**) output microvessel image of HDMI. (2) HDMI morphometric analysis with morphological filtering and vessel segmenting steps that leads to (**B**) conversion of the microvasculature image into a binary image, (**C**) skeleton showing the network of vessels, and (**D**) overlay of the skeleton on the binary vessel image with identified branching points. The extracted biomarkers are used to differentiate malignant melanoma from benign nevus. Brief descriptions and calculations of four important quantitative biomarkers—τ, MD, mvFD, and BA—are shown [14,15,16].

For each choroidal tumor, a region of interest (ROI) was defined based on the B-mode ultrasound images. The segmented lesion ensured that all vessel quantification steps were limited to the specified ROI. The HDMI image was converted to a binary image, and the full skeleton of the microvessel network was constructed for morphological parameter quantification [16], as shown in Figure 2.

The methods for obtaining HDMI images and quantitative biomarkers have been previously described [15,16,17]. Briefly, vessel density (VD) was defined as the proportion of vessel area with blood flow over the total area measured [16]. Additional biomarkers included the number of vessel segments (NV), the number of branch points (NB) defined as a common point connected to three or more vessel segments [15,16], and vessel diameter (D) [16]. Vessel tortuosity (*τ*) was defined as the ratio between the actual path length of a meandrous vessel and the linear distance between the two endpoints. Murray’s deviation (MD) indicates a diameter mismatch, defined as the deviations from Murray’s Law, detailed in [17]. Microvessel fractal dimension (mvFD) is a unit-less geometrical feature indicating the structural complexity of a vascular network [17,26]. Bifurcation angle (BA) was defined as the angle between two daughter vessels and has also been used as a distinguishing biomarker of malignancy [17]. For tortuosity, D, MD, and BA, we considered both the mean and maximum of all measures for a given participant (i.e., $\tau_{mean},\;\tau_{max},\;D_{mean}$, $D_{max}$, ${MD}_{mean}$, ${MD}_{max}$, ${BA}_{mean}$, and ${BA}_{max}$).

### 2.6. Statistical Analysis Methods

Quantitative variables were summarized as mean ± standard deviation (SD) and ranges, while nominal variables were summarized as counts and percentages. A non-parametric Wilcoxon rank-sum test was used to test differences in distributions of quantitative biomarkers between malignant and benign tumors. A two-sided *p*-value < 0.05 was considered statistically significant, and reported p-values were left unadjusted for multiple testing. For corresponding measures of discrimination, we additionally estimated the area under the receiver operating characteristic curve (AUC) and corresponding 95% confidence interval (CI). In the case of ${MD}_{mean}$, ${MD}_{max}$, ${BA}_{mean}$, and ${BA}_{max}$, there is potential structural missingness for individuals for whom there were no vascular branch points to calculate these biomarkers (i.e., NB = 0). To jointly analyze associations of both quantitative values and measurability, multivariable Firth logistic regression models were applied with the linear predictor defined as $\beta_{0}+\beta_{1}X_{1}+\beta_{2}{X_{1}X}_{2}$, where $X_{1}$ is the binary indicator of measurability (0 = missing, 1 = observed) and $X_{2}$ is the quantitative biomarker value. Note that $\beta_{2}$ only applies when the measurement is observed; thus, $X_{2}$ can be arbitrarily defined for instances of missing values. The effects $\beta_{1}$ and $\beta_{2}$ were jointly assessed per biomarker using 2 degree-of-freedom likelihood ratio tests (LRT). Previously mentioned statistical analyses were performed using RStudio version 6.0.421 (Boston, MA, USA) with R version 4.3.0 (R Core Team, Vienna, Austria). Pearson’s correlations among HDMI biomarkers were estimated using Python version 3.10.12 (Wilmington, DE, USA), and only pair-wise complete data were included in this analysis.

## 3. Results

Out of the 36 choroidal tumors from 36 participants (21 male and 15 female), 21 (58%) were malignant choroidal melanomas and 15 (42%) were benign choroidal nevi. The diagnosis was made by a fellowship-trained ocular oncologist using clinical examination and multimodal ophthalmic imaging. Among the twenty-one male participants, fourteen had malignant melanomas and seven had benign nevi; among the fifteen females, seven had malignant melanomas and eight had benign nevi. Participant age was 60.48 ± 15.75 years (range: 30 to 86) in the malignant participant group and 67.47 ± 14.31 (range: 34 to 89) among participants with benign masses. All participants were of non-Hispanic white race and ethnicity. Table 1 summarizes the demographic characteristics and clinical imaging features of all participants. 

### 3.1. Clinical Multimodal Ophthalmic Imaging Features

Tumor thicknesses for malignant melanoma and benign nevus were 3.81 ± 2.63 mm (range: 0.91 mm to 11.3 mm) and 1.70 ± 0.40 mm (range: 1.20 mm to 3.19 mm), respectively, with a *p* value of 0.0005. Clinical ultrasound features such as low internal reflectivity and subretinal fluid were reported in ten and five malignant melanomas, respectively. Vascular irregularity was reported in clinical OCTA of five melanomas and nine nevi. The FA of tumors showed orange pigment in fifteen melanomas and eight nevi.

### 3.2. Microvessel Visualization and Quantification

**Figure 3:**
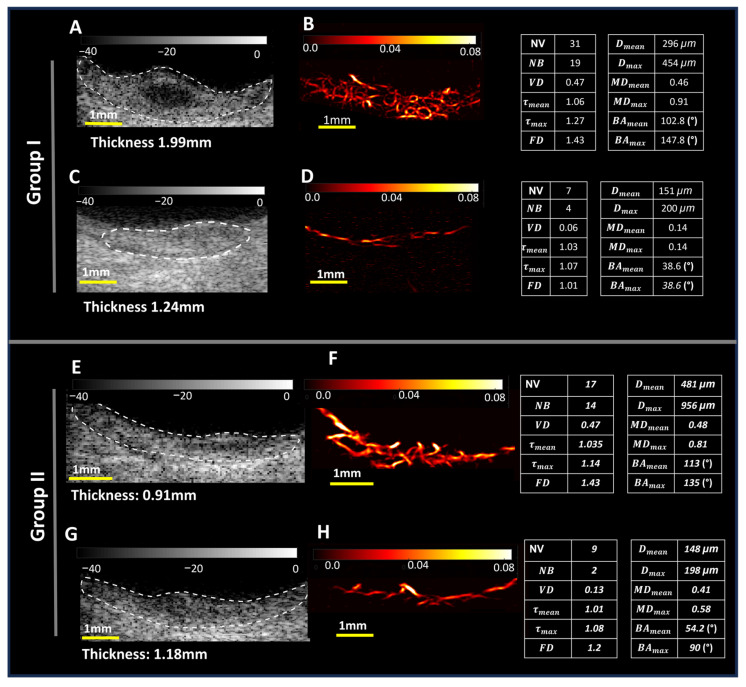
Two groups of small choroidal tumors with thickness less than 2mm. Group I: B-mode images (**A**,**C**) and HDMI images (**B**,**D**). In this group, (**A**,**B**) are images from a 34-year-old male participant with diagnosis of choroidal melanoma with tumor thickness of 1.99mm; (**C**,**D**) images from a 52-year-old female participant with choroidal nevus with tumor thickness 1.24mm; the corresponding quantified HDMI biomarkers are indicated on the right side of the figure. Group II: B-mode images (**E**,**G**) and HDMI images (**F**,**H**). In this group, (**E**,**F**) are images for choroidal melanoma of a 63-year-old male participant with choroidal melanoma with thickness of 0.91mm; (**G**,**H**) images from a 72-year-old female participant with choroidal nevus with tumor thickness 1.18 mm. Tables including the corresponding quantified HDMI biomarkers are indicated on the right side of the figure.

We present two sets of small benign and malignant choroidal melanocytic tumors (nevus and melanoma) based on the most important clinical ultrasound tumor dimension, tumor thickness, for visual comparison along with metric values of quantitative biomarkers of HDMI (Figure 3). In group I, the conventional B-mode ultrasound and HDMI images with their corresponding HDMI quantitative biomarkers are shown in Figure 3A–D. The thicknesses of the tumors in this set are approximately 1.99 mm and 1.24 mm for the malignant choroidal melanoma and benign nevus, respectively. As seen in Figure 3B,D, HDMI provided high-resolution images of tumor microvessels without the use of contrast agents, which is evident under visual inspection, showing hypervascularity with increased complexity in choroidal melanoma compared to benign choroidal nevus with noticeably fewer microvessels and a less complex morphology. All quantified HDMI biomarkers shown on the side of this group indicate higher values in choroidal melanoma compared to benign nevus. The thicknesses of the tumors in group II are approximately 0.91 mm and 1.18 mm for the malignant choroidal melanoma and benign nevus, respectively. Choroidal melanoma, shown in Figure 3E,F, is the transformation of a previously known choroidal nevus. Complexity and increased vascularity in visual inspection, as well as higher values in HDMI metrics, are seen in melanoma compared to nevus.

**Figure 4:**
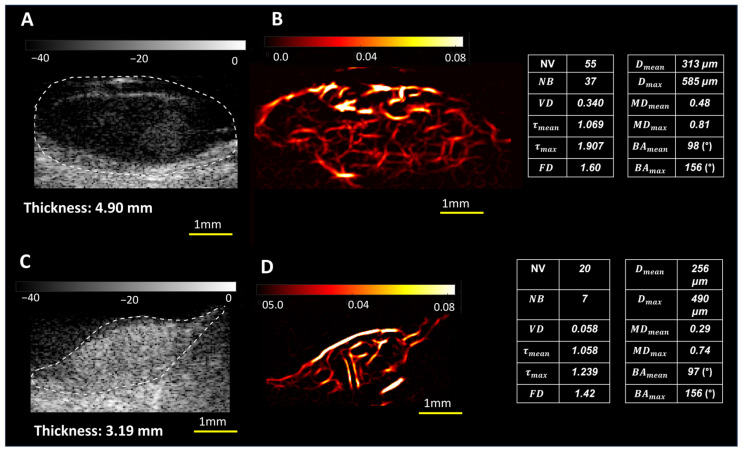
B-mode (**A**,**C**) and HDMI (**B**,**D**) images for choroidal melanoma and hemangioma. In this group, (**A**,**B**) are images from a 30-year-old female participant with a diagnosis of choroidal melanoma with tumor thickness of 4.90mm; (**C**,**D**) are images from of a 63-year-old female participant with benign choroidal hemangioma with a tumor thickness of 3.19mm. The corresponding quantified HDMI biomarkers are shown on the right side of the figure.

Figure 4 presents a set of two larger choroidal tumors with thicknesses of malignant melanoma (thickness: 4.90 mm) and benign hemangioma (3.19 mm). While benign hemangioma is excluded from the analysis because of the focus of the study on choroidal melanoma vs. nevus, we selected hemangioma for a case comparison because of the size and hypervascular nature of this tumor. Visually, hypervascularity with comparable vessel density can be seen in both melanoma and hemangioma. However, higher values of other HDMI morphological metrics—tortuosity, NB, NV, NB, MD, mvFD, and BA—are seen in malignant melanoma compared to benign hemangioma.

### 3.3. Statistical Analysis

Table 2 and Table 3 summarize the quantitative HDMI biomarkers’ distributions and analysis results. Out of the eight quantitative HDMI biomarkers analyzed using the Wilcoxon rank-sum test, six (75%) showed significantly higher values in the malignant choroidal melanomas compared to the benign nevi, including NV (AUC = 0.79, 95% CI = [0.47,0.93]; *p* = 0.003), NB (AUC = 0.78, 95% CI = [0.39,0.94]; *p* = 0.003), τ_max_ (AUC = 0.82, 95% CI = [0.64,0.95]; *p* = 0.001), $D_{max}$ (AUC = 0.71, 95% CI = [0.50,0.88]; *p* = 0.003), VD (AUC = 0.72, 95% CI = [0.51,0.88]; *p* = 0.03), and mvFD (AUC = 0.81, 95% CI = [0.63,0.95]; *p* = 0.001). Similarly, the logistic regression analyses accounting for structural missingness identified significant associations with ${BA}_{mean}$ (LRT *p* = 0.002)$,{BA}_{max}$ (*p* = 0.002), ${MD}_{mean}$ (*p* = 0.002), and ${MD}_{max}$ (*p* = 0.002). These HDMI biomarkers (${MD}_{mean}$, ${MD}_{max}$, ${BA}_{mean}$, and ${BA}_{max}$) each had seven missing values for the benign group (47%) due to the absence of a branch. No missing values were reported for the malignant group.

**Figure 5:**
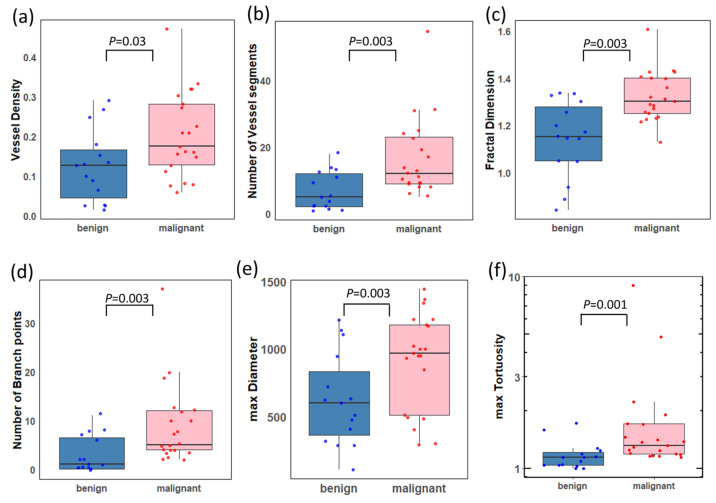
Boxplots of the distributions of significant HDMI biomarkers for benign and malignant choroidal tumors. (**a**) Vessel density (VD), (**b**) number of vessel segments (NV), (**c**) microvessel fractal dimension (mvFD), (**d**) number of branch points (NB), (**e**) maximum diameter ($D_{max}$), and (**f**) maximum tortuosity ($\tau_{max}$) (plotted on a log scale). A two-sided *p*-value < 0.05 was considered statistically significant. Benign (*n* = 15); malignant (*n* = 21).

Figure 5 depicts the boxplots for the significant HDMI biomarkers. All HDMI biomarkers demonstrated higher median values in the malignant group compared to the benign group.

**Figure 6:**
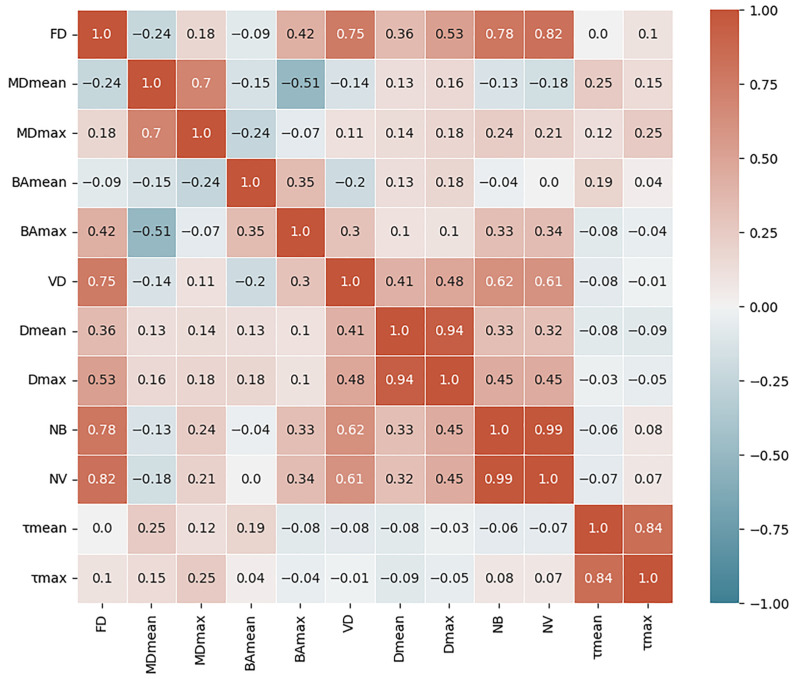
Pearson correlation matrix of the HDMI biomarkers (for pairwise complete observations). Cyan cells correspond with a negative correlation, and red cells correspond with a positive correlation.

In Figure 6, we present the Pearson correlation matrix to assess the interrelationships among the HDMI biomarkers. mvFD was found to be strongly correlated with VD (ρ = 0.75), NB (ρ = 0.78), and NV (ρ = 0.82), indicating that as mvFD increases, VD, NB, and NV increase as well. Tortuosity values (τ_mean_,τ_max_) were weakly correlated with all the other parameters, indicating that they may represent independent biomarker information.

**Figure 7:**
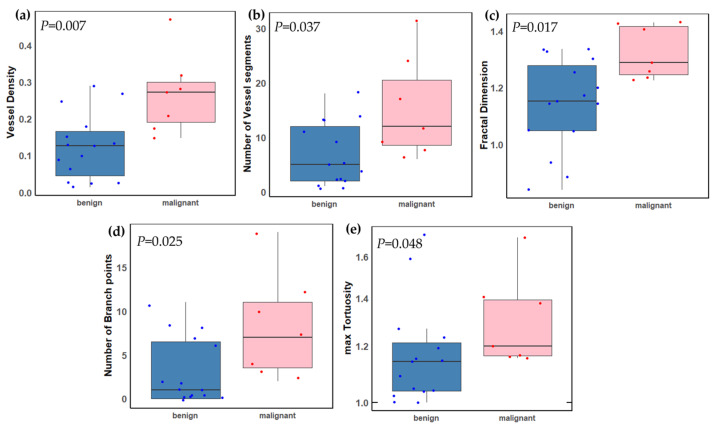
Boxplots of the distributions of significant qHDMI biomarkers in a subset of benign and malignant choroidal tumors based on thickness ≤ 2.5mm. (**a**) Vessel density (VD), (**b**) number of vessel segments (NV), (**c**) microvessel fractal dimension (mvFD), (**d**) number of branch points (NB), and (**e**) maximum tortuosity ($\tau_{max}$). A two-sided *p*-value < 0.05 was considered statistically significant. Benign (*n* = 15); malignant (*n* = 7).

One potential confounding factor in our data is tumor thickness, as malignant lesions were significantly thicker than benign lesions (Wilcoxon *p* = 0.0005). To assess the robustness of our results, we performed sensitivity analyses restricted to tumors with thicknesses of 2.5 mm or less (including all fifteen benign lesions and seven malignant lesions). We found no significant difference in size between this subgroup of melanoma and the nevi group (*p* = 0.778). However, among the six previously significant qHDMI parameters, five remained significantly different by malignancy status in this tumor subset (Table 4, Figure 7).

## 4. Discussion

This study investigated the discriminatory potential of the quantitative biomarkers of contrast-free qHDMI between a malignant choroidal melanoma and benign choroidal nevus. Our findings show that qHDMI biomarkers VD, NV, NB, maximum diameter ($D_{max}$), maximum tortuosity ($\tau_{max}$), and mvFD showed significant distinctions between the malignant choroidal melanoma and benign nevus groups. Another important finding of this study is that qHDMI was able to separate very small melanomas, as small as 0.91 mm, from small nevi, as shown in Figure 3. One potential confounding factor in our study is the tumor thickness, as malignant lesions were significantly thicker than benign lesions (Wilcoxon *p* = 0.0005). While the diagnosis of large choroidal melanomas thicker than 5 mm is less challenging, they metastasize at this later stage. However, the ultimate goal is to distinguish choroidal melanomas from nevi at an early stage as they share similar ophthalmoscopic appearances when small. In a subset size analysis, our study found no significant difference in size for early-stage melanomas 2.5 mm or less in thickness (Wilcoxon *p* = 0.778). However, five of the qHDMI biomarkers remained significantly different by malignancy status for this subset of small lesions. Therefore, our findings underscore the potential effectiveness of quantitative HDMI biomarkers in distinguishing early-stage melanomas from nevi (smaller than 3mm in thickness), which is in agreement with previous studies on detecting very small and early-stage breast and liver cancers [18,19,20,23].

Malignant transformation of an existing nevus as well as de novo cases of choroidal melanoma are accompanied by the formation of new aberrant and complex vessels [7]. Such newly formed tumor vessels are structurally abnormal, showing an increased diameter, abnormal branching pattern, irregularity, and complexity [9]. Currently, there are no noninvasive tools for imaging and quantifying angiogenesis in choroidal melanoma. A previous study using a contrast-enhanced color Doppler aimed to assess the tumor vasculature in uveal melanomas, but was unable to differentiate normal vessel structures from those of the tumor [27]. The current study, however, provided high-resolution images of tumor microvessels without the use of contrast agents and, more importantly, the quantitative morphological biomarkers were differentiable between a malignant uveal melanoma and a nevus.

Until now, only preclinical studies using ULM have been explored for imaging microvessels of choroidal melanoma [28]. In the current study, HDMI provided high-resolution images of the microvessel network of choroidal tumors, and the majority of quantitative HDMI biomarkers showed significant differences between choroidal melanoma and a nevus. The most important finding in our study is that qHDMI was able to capture and quantify the microvasculature features in early-stage choroidal melanomas. Distinguishing choroidal melanoma at an early stage from a nevus can be challenging, as they share similar clinical appearances when small [3]; thus, early detection and treatment is perhaps the most effective way to prevent metastasis in a cancer where approximately 50% of patients will ultimately develop metastatic disease [29]. However, standard-of-care treatments using radiotherapy carry a high risk for vision loss, leading to frequent observation in cases of indeterminate choroidal melanocytic lesions to watch for documented growth prior to treatment [30]. This practice, driven by the somewhat subjective nature of choroidal melanoma diagnosis, even amongst ocular oncologists, could lead to treatment delays and increased metastatic risk. Thus far, there is no single or objective modality to successfully distinguish a choroidal nevus from melanoma; so, the technique described in this study has the potential to make an important clinical impact.

In the current study, higher values of VD, NV, and NB in malignant melanomas compared to benign nevi are supported by similar findings observed in cancer detection for patients with tumors in the breast [18], liver [23], thyroid nodules [22], and lymph nodes [21]. Also, maximum tortuosity and maximum diameter showed higher values in the malignant group compared to the benign group and empirically exhibited higher discrimination than mean tortuosity and mean diameter. Our findings are supported by the fact that increased tortuosity and vessel diameter are more prevalent on the periphery compared to the center of the growing malignant tumor. This fact suggests that the use of maximum vessel diameter and tortuosity may provide a better diagnostic value than averaging the diameter of the vessels [9].

We found higher values of mvFD in malignant choroidal melanoma, which is consistent with the fact that microvessel complexity measured by mvFD is higher in malignant tumors and its measurement may provide important diagnostic and prognostic information [26,31]. The present study also found vessel density to be higher in malignant tumors. Similar findings have been reported with the use of contrast agents [13]. Vessel density was found to be an important prognostic factor for survival in patients with choroidal melanoma, indicating that higher vessel density in choroidal melanoma is associated with hepatic metastasis and higher mortality rates [32,33,34].

The current study had limitations that warrant mention. The sample size was insufficient to employ more sophisticated multivariable statistical analyses and for the development of predictive models. Plans for future studies will include a larger cohort of participants to evaluate the performance of HDMI biomarkers more precisely for early detection of choroidal melanomas while additionally controlling for potential confounding factors (e.g., tumor thickness). There is also the possibility of data degradation due to voluntary and involuntary eye movement during data acquisition. The future plan will include utilizing and expanding the motion correction algorithms [35,36] to reduce potential motion artifacts or to use deep learning methods [37,38] for the correction of motion artifacts. Lastly, future work should include complementary quantitative three-dimensional HDMI imaging [20] to overcome the limitations of our 2D imaging for morphological features.

## 5. Conclusions

In conclusion, our study demonstrated the feasibility of quantitative HDMI for differentiation of choroidal melanoma and choroidal nevus in tumors with a thickness smaller than 2mm, indicating the potential of this technique for early detection of choroidal melanoma. In the future, we will focus more on the detection of early-stage choroidal melanoma and treatment monitoring as well as investigating the possibility of discriminating between other types of ocular tumors.

## 6. Patents

US Patent 11,213,278 and EP 3 634 238 B1, JP 7139357 B2.

## Figures and Tables

**Table 1:** Participant demographics, clinical and lesion characteristics, included in this study.

Characteristics	Malignant (21)	Benign (15)
**Demographics**		
Sex (male)	14 (67%)	7 (47%)
Sex (female)	7 (33%)	8 (53%)
Age (years)	60.48 ± 15.75	67.47 ± 14.31
White non-Hispanic (race and ethnicity)	21 (100%)	15 (100%)
**Clinical multimodal imaging features**		
Tumor thickness, mm (US)	3.81 ± 2.63	1.70 ± 0.40
Tumor basal diameter, mm (US)	10.45 ± 3.82	7.78 ± 1.50
Vascular irregularity over lesion (OCTA)	5 (24%)	9 (60%)
Low internal reflectivity (US)	10 (48%)	5 (33%)
Subretinal fluid (US)	5 (24%)	0 (0%)
Orange pigment (FA)	15 (71%)	8 (53%)

**Table 2:** Quantitative HDMI biomarkers for benign and malignant choroidal tumors and their corresponding *p*-values.

HDMI Biomarkers	Malignant *n =* 21	Benign *n =* 15	*p*-Value	AUC [95% CI]
VD	0.20 ± 0.11	0.12 ± 0.09	0.03	0.72 [0.51, 0.88]
$D_{mean}$(µm)	459.89 ± 174.46	381.83 ± 176.38	0.39	0.59 [0.39, 0.78]
$D_{max}$(µm)	908.56 ± 359.09	624.32 ± 341.80	0.003	0.71 [0.50, 0.88]
NV	16.57 ± 11.84	6.73 ± 5.74	0.003	0.79 [0.47, 0.93]
mvFD	1.32 ± 0.11	1.14 ± 0.16	0.002	0.81 [0.63, 0.95]
NB	8.90 ± 8.33	3.07 ± 3.81	0.003	0.78 [0.39, 0.94]
$\tau_{mean}$	1.14 ± 0.21	1.05 ± 0.03	0.14	0.65 [0.45, 0.83]
$\tau_{max}$	1.91 ± 1.81	1.18 ± 0.21	0.001	0.82 [0.64, 0.95]

Note: VD, D, NV, mvFD, NB, and *τ* refer to vessel density, diameter, number of vessel segments, fractal dimension, number of branch points, and tortuosity, respectively.

**Table 3:** Quantitative HDMI biomarker for benign and malignant choroidal tumors for branching-based biomarkers and their corresponding *p*-values.

HDMI Branching-Based Biomarkers.	Malignant *n =* 21		Benign *n =* 15		*p*-Value
	N w/NB > 0 (%)	Mean ± SD *	N w/NB > (%)	Mean ± SD *	
${BA}_{mean}$ (°)	21 (100%)	99.53 ± 17.72	8 (53%)	99.74 ± 32.92	0.002 †
${BA}_{max}$ (°)	21 (100%)	141.25 ± 28.57	8 (53%)	140.28 ± 35.24	0.002 †
${MD}_{mean}$	21 (100%)	0.40 ± 0.18	8 (53%)	0.40 ± 0.18	0.002 †
${MD}_{max}$	21 (100%)	0.70 ± 0.26	8 (53%)	0.66 ± 0.22	0.002 †

Note: † These *p*-values were obtained from the likelihood test of the multivariable logistic regression analysis. BA and MD refer to bifurcation angle and Murray’s deviation, respectively. * Mean+-SD are based on a subset of observations with observed values.

**Table 4:** Quantitative HDMI biomarkers for a subset of tumors with thickness of 2.5 mm or less and their corresponding *p*-values.

	Malignant *n* = 7	Benign, *n* = 15	*p*-Value
Tumor thickness	1.74 ± 0.45)	1.70 ± 0.41	0.778
**HDMI biomarkers**			
VD	0.27 ± 0.11	0.12 ± 0.09	0.007
$D_{mean}$ (µm)	405.48 ± 254.82	381.83 ± 176.38	>0.99
$D_{max}$ (µm)	752.63 ± 499.35	624.32 ± 341.80	0.724
NV	15.29 ± 9.27	6.73 ± 5.74	0.037
mvFD	1.32 ± 0.10	1.14 ± 0.16	0.017
NB	8.14 ± 6.04	3.07 ± 3.81	0.025
$\tau_{mean}$	1.07 ± 0.04	1.05 ± 0.03	0.622
$\tau_{max}$	1.31 ± 0.20	1.18 ± 0.21	0.048

Note: VD, D, NV, mvFD, NB, and *τ* refer to vessel density, diameter, number of vessel segments, fractal dimension, number of branch points, and tortuosity, respectively. *p*-values in bold indicate statistical significance.

## Data Availability

The data that support the findings of this study are available from the corresponding author upon reasonable request. The requested data may include figures that have associated raw data. Because the study was conducted on human volunteers, the release of patient data may be restricted by Mayo policy and needs special request. The request can be sent to Karen A. Hartman, MSN, CHRC|Administrator—Research Compliance|Integrity and Compliance Office|Assistant Professor of Health Care Administration, Mayo Clinic College of Medicine & Science|507-538-5238|Administrative Assistant: 507-266-6286|hartman.karen@mayo.edu Mayo Clinic|200 First Street SW|Rochester, MN 55905|mayoclinic.org (accessed on 14 January 2024). We do not have publicly available accession codes, unique identifiers, or web links.
